# Post-stroke dizziness in anterior vs. posterior circulation ischemic stroke

**DOI:** 10.3389/fneur.2026.1742461

**Published:** 2026-02-26

**Authors:** Sang Hee Ha, Gayoung Park, Bum Joon Kim, Jun Young Chang, Dayoung Seo, Dong-Wha Kang, Sun U. Kwon, Jong S. Kim, Eun-Jae Lee

**Affiliations:** 1 Department of Neurology, Gil Medical Center, Gachon University, Incheon, Republic of Korea; 2 Department of Neurology, Asan Medical Center, University of Ulsan College of Medicine, Seoul, Republic of Korea; 3 Department of Neurology, Gangneung Asan Hospital, University of Ulsan, Gangneung, Gangwon-do, Republic of Korea; 4 Translational Biomedical Research Group, Asan Medical Center, University of Ulsan, Seoul, Republic of Korea

**Keywords:** anterior circulation stroke, beck depression inventory, post stroke dizziness, posterior circulation stroke, Spielberger State-Trait Anxiety Inventory

## Abstract

**Background:**

Dizziness in anterior circulation stroke (ACS) has not been well characterized. We aimed to examine the frequency of dizziness and its associated factors in ACS, and to compare these findings with posterior circulation stroke (PCS).

**Methods:**

We prospectively enrolled consecutive patients with acute ischemic stroke from July 2021 to July 2022, categorized into ACS and PCS groups. The presence of new-onset dizziness was assessed within 7 days of stroke onset in clinically stable patients, excluding those with severe deficits that precluded survey completion. Clinical variables, depressive symptoms (Beck Depression Inventory), anxiety (State–Trait Anxiety Inventory), and neuroimaging findings were collected. Multivariable logistic regression analyses were performed to identify factors independently associated with dizziness.

**Results:**

Among 169 patients (98 ACS, 71 PCS), dizziness was reported in 45.9% of patients with ACS and 60.6% of those with PCS. In the ACS group, the presence of cerebral microbleeds [adjusted odds ratio (aOR) = 3.19, 95% confidence interval (CI) 1.09–9.32, *p* = 0.034] or a higher number of microbleeds (aOR = 2.38, 95% CI 1.10–5.15, *p* = 0.026) were independently associated with dizziness. In the PCS group, dizziness was independently associated with medullary or cerebellar lesions (aOR = 3.13, 95% CI 1.01–9.74, *p* = 0.048).

**Conclusion:**

Dizziness was common in patients with ACS, with a frequency comparable to that in PCS. The absence of an association with depressive or anxiety symptoms, together with the link to cerebral microbleeds, suggests that dizziness in ACS may reflect underlying structural or vascular mechanisms, warranting greater clinical attention.

## Introduction

Dizziness is one of the most frequent and challenging symptoms encountered in clinical neurology, encompassing a broad spectrum of sensations including vertigo, presyncope, disequilibrium, and nonspecific subjective sensations (1). Within the context of cerebrovascular disease, stroke is a critical etiology to identify, accounting for approximately 5–10% of all acute vestibular presentations in emergency settings (2–5).

Traditionally, both clinical teaching and research have focused almost exclusively on posterior circulation stroke (PCS) as the primary vascular cause of dizziness. This emphasis reflects the direct involvement of the vestibular axis—comprising the brainstem nuclei, the cerebellum, and their associated cranial nerves—which, when infarcted, typically produce overt and often dramatic vestibular symptoms (6, 7). Consequently, current diagnostic algorithms and management guidelines for post-stroke dizziness are heavily weighted toward PCS-related mechanisms (8), and dizziness occurring in the context of anterior circulation stroke (ACS) has often been regarded as atypical, nonspecific, or secondary, receiving less systematic clinical attention.

However, patients with ACS may also experience dizziness. Emerging evidence suggests that the human vestibular system is more distributed, involving a complex network of cortical and subcortical structures (9). Specifically, the parietal cortex and the insular are considered central structures of the human vestibular cortical network, playing a pivotal role in integrating multimodal sensory information to maintain spatial orientation (9, 10). Infarctions in these ACS territories can disrupt these higher-order processing centers, leading to dizziness even in the absence of classical brainstem or cerebellar involvement (10). Importantly, dizziness related to ACS may lack clear localizing neurological signs and may not conform to classical vestibular syndromes, making symptom attribution more challenging in routine clinical practice.

Furthermore, dizziness in ACS may arise from direct involvement of central vestibular pathways as well as secondary neuropsychiatric sequelae, including post-stroke depression and anxiety (11–13). Given the nonspecific nature of dizziness, emotional symptoms may influence symptom perception or reporting, potentially confounding the distinction between stroke-related mechanisms and affective contributions. Nevertheless, systematic comparisons of dizziness between ACS and PCS remain scarce, and the specific determinants of dizziness in ACS patients are poorly characterized. Most previous studies have focused on posterior circulation lesions, specific symptom subtypes such as vertigo, or retrospective cohorts, limiting direct comparisons between stroke territories (9). In addition, the potential contribution of mood-related symptoms has rarely been evaluated in prospective stroke cohorts using validated instruments, especially with respect to differences between anterior and posterior circulation strokes.

In this prospective study, we aimed to address these gaps by characterizing the frequency of dizziness and its associated clinical and neuroimaging features in patients with ACS and by directly comparing these findings with those observed in PCS. By utilizing validated instruments, including the Beck Depression Inventory (BDI) and the Spielberger State–Trait Anxiety Inventory (STAI) (14), we sought to disentangle stroke-related factors from emotional influences on dizziness perception. Ultimately, this study aims to clarify the clinical characteristics and imaging correlates of dizziness across different stroke territories, thereby improving recognition and territory-specific management of this frequently overlooked symptom in patients with acute ischemic stroke.

## Materials and methods

### Patient selection

This study involved a prospective analysis of consecutive patients who presented with an acute ischemic stroke within 7 days of symptom onset at Asan Medical Center (Seoul, South Korea) between July 2021 and July 2022. We excluded patients who exhibited severe neurological deficits that precluded completion of study questionnaires. The patients with the following conditions were excluded: (1) altered mental status (drowsiness or more severe decreased consciousness, or disorientation and confusion as diagnosed by attending neurologists); (2) severe motor weakness (Medical Research Council score ≤3); (3) significant hemispheric cortical signs, such as aphasia, neglect, or visual disturbances (including diplopia or hemianopia); (4) severe dementia (14); (5) a previous modified Rankin score greater than 3; (6) no acute lesion on diffusion-weighted imaging (DWI); and (7) active systemic diseases (such as infection or active cancer) (15). Eligible patients completed the State–Trait Anxiety Inventory (STAI) and Beck Depression Inventory (BDI) questionnaires after neurological stabilization. We further excluded patients with lesions involving both anterior and posterior circulations, absence of acute lesions on imaging, or incomplete questionnaire data.

The study was approved by the Institutional Review Board of Asan Medical Center (approval number: 2021–0901), and written informed consent was obtained from all participants. All study procedures were conducted in accordance with the Declaration of Helsinki.

### Clinical and imaging assessments

Demographic information and clinical variables were collected. Neurological examinations at enrollment were conducted by an experienced neurologist. The cause of stroke was categorized according to the Trial of Org 10,172 in Acute Stroke Treatment classification system (16). Furthermore, neurological deficits were evaluated using the National Institutes of Health Stroke Scale (NIHSS).

All patients underwent MRI on a 3.0 T Philips scanner (Philips Healthcare, Eindhoven, Netherlands), including DWI and MR angiography. The locations of acute lesions were assessed on DWI and classified as follows: PCS lesions, including those in the cerebellum [superior cerebellar, anterior inferior cerebellar, and posterior inferior cerebellar (PICA) artery territories], medulla oblongata, pons, midbrain, thalamus, and medial temporo-occipital areas; ACS lesions, including those in the frontal cortex/subcortex, parietal cortex/subcortex, temporal cortex/subcortex, deep structures, and insula. Multiple lesion locations were recorded if a patient had ischemic lesions in more than one region, with each classified as a separate variable; thus, a single patient could contribute multiple lesion sites (5). Dorsal brainstem stroke was defined as involvement of the dorsal medulla oblongata or the tegmentum of the pons and midbrain, regions implicated in the central vestibular pathway (17). Lesion locations were determined by consensus between two stroke experts (SH Ha and E-J Lee) who were blinded to clinical information.

Small vessel disease burden was evaluated on fluid-attenuated inversion recovery (FLAIR) imaging. White matter hyperintensities (WMHs) were defined as periventricular hyperintense lesions on T2-weighted FLAIR and graded according to the modified Fazekas scale: 0 = absent; 1 = pencil-thin lining; 2 = halo ≥5 mm in thickness; 3 = irregular WMHs extending into the deep white matter. Patients were subsequently dichotomized into two groups: grades 0–1 versus 2–3 (18). Cerebral microbleeds (CMBs) were defined as small perivascular hemosiderin deposits, appearing as rounded, homogeneous, hypointense lesions on T2*-weighted gradient-recalled echo or susceptibility-weighted MRI, and both lobar and deep CMBs were included. Lacunes were defined as chronic small cavities, presumed to represent the healed stage of a lacunar infarct (19).

### Questionnaire

**Figure 1:**
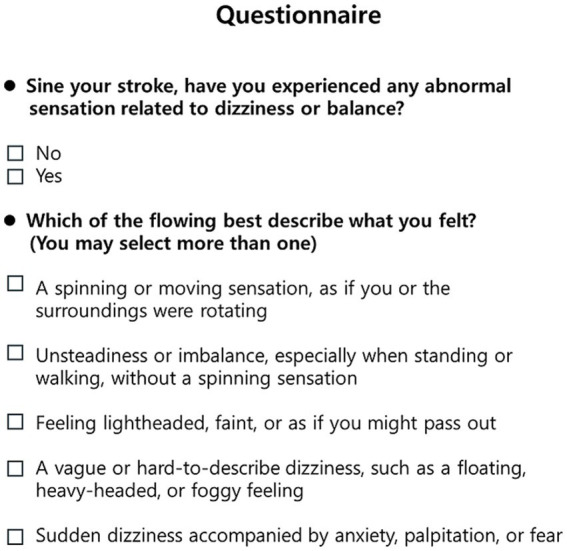
Dizziness questionnaire.

We investigated the occurrence of new-onset dizziness following stroke, focusing on whether patients experienced any dizziness symptom regardless of its qualitative presentation (20) (Figure 1). Specifically, dizziness was assessed using a structured patient interview; patients were first asked whether they had experienced any abnormal sensations related to dizziness or balance since stroke onset. If present, they were asked to select symptom descriptors that best characterized their experience, including spinning sensation, unsteadiness or imbalance, lightheadedness or near-faintness, and vague or difficult-to-describe dizziness. The presence of dizziness was defined as a positive response to any of these symptom descriptors. This broad, symptom descriptor–based definition was adopted to minimize under-detection of post-stroke dizziness by encompassing both vertiginous and non-vertiginous manifestations. To ensure clinical consistency, the presence of dizziness was verified through structured interviews conducted by two trained neurological nurse specialists (GY Park and DY Seo), who were blinded to all clinical information. In cases where the nature of the symptoms was ambiguous, the final determination of whether the patient was symptomatic was made by consensus between the two raters.

We additionally administered standardized anxiety and depression questionnaires, given that dizziness is often nonspecific and may be influenced by emotional symptoms. The State–Trait Anxiety Inventory (STAI), a validated instrument for assessing trait and state anxiety, includes 20 items for trait anxiety, with scores ranging from 20 to 80 (higher scores indicating greater anxiety) (21). The Beck Depression Inventory (BDI) is a 21-item self-report scale for depressive symptoms, with responses from 0 (not at all) to 3 (very much) and total scores ranging from 0 to 63 (22).

### Statistical analysis

Baseline characteristics were compared between ACS and PCS patients. Continuous variables were expressed as mean ± standard deviation or median [interquartile range] after testing for normality using the Shapiro–Wilk test. Comparisons between groups were performed using Student’s t-test or the Mann–Whitney U test for continuous variables and Fisher’s exact test for categorical variables. To identify independent factors associated with dizziness, we performed multivariable logistic regression analyses. Variables that reached a entry threshold of *p* < 0.1 in univariable analysis, as well as age, were included as covariates. For the ACS group, two distinct models were constructed to avoid multicollinearity between the presence and the count of cerebral microbleeds. All analyses were performed using IBM SPSS Statistics, version 21.0 (IBM Corp., Armonk, NY, USA). Statistical significance was set at *p* < 0.05.

## Results

### Baseline characteristics

**Figure 2:**
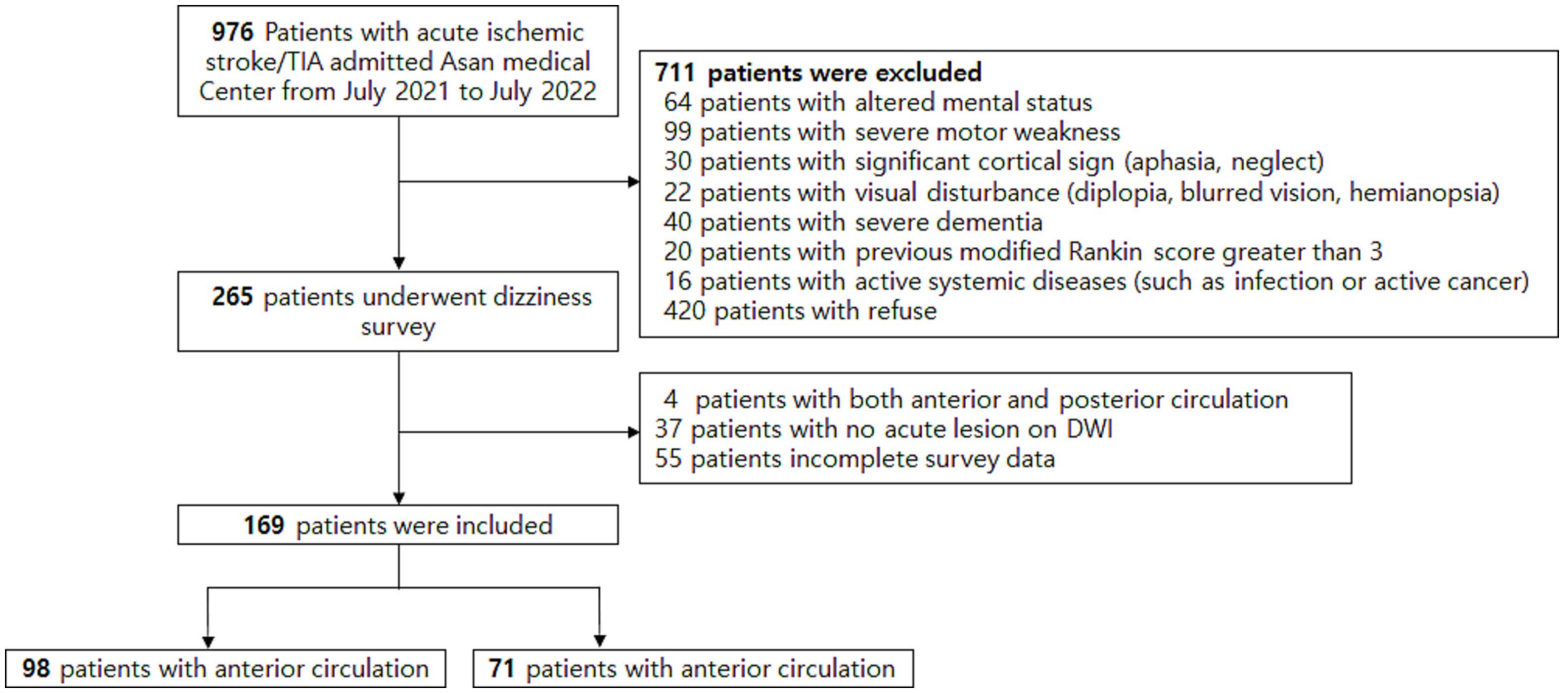
Study flow chart. mRS, modified Rankin Scale; TIA, transient ischemic attack.

During the study period, a total of 976 patients with acute ischemic stroke were admitted to our center, and 265 patients completed the survey (Figure 2). All questionnaires were administered within a median of 1 day (range, 1–3 days) after stroke onset. Compared with the non-survey group (*n* = 711), survey completers were younger (64 ± 13 vs. 68 ± 14 years; *p* < 0.001) and had lower initial NIHSS scores [2 (0–4) vs. 3 (1–8); *p* < 0.001] (Supplementary Table S1). Of the surveyed patients, 4 with both anterior and posterior circulation stroke, 37 without an acute lesion on DWI, and 55 with incomplete survey data were excluded. The final analysis included 169 patients (17.3%), comprising 98 with ACS (58.0%) and 71 with PCS (42.0%).

The demographic and baseline characteristics are summarized in Table 1. The mean age was 63 ± 13 years, 112 patients (66.3%) were male, and the median initial NIHSS score was 2 (0–4). The mean BDI and STAI scores were 11 ± 9 and 41 ± 8, indicating mild depressive symptoms and moderate anxiety (21, 22). No significant differences in baseline characteristics were observed between the ACS and PCS groups, including stroke etiology (TOAST classification) and neurological severity (NIHSS score).

**Table 1:** Baseline characteristics of patients with anterior and posterior circulation stroke.

	Total *N* = 169	ACS *n* = 98	PCS *n* = 71	*p-*value
Age (years)	63 ± 13	62 ± 14	64 ± 12	0.865
Male sex	112 (66.3)	67 (68.4)	45 (63.4)	0.499
Hypertension	103 (60.9)	57 (58.2)	46 (64.8)	0.384
Diabetes mellitus	50 (29.6)	25 (25.5)	25 (35.2)	0.173
Hyperlipidemia	86 (50.9)	49 (50.0)	37 (52.1)	0.786
Atrial fibrillation	33 (19.5)	21 (21.4)	12 (16.9)	0.464
Smoking	65 (38.5)	40 (40.8)	25 (35.2)	0.460
History of previous stroke	43 (25.4)	23 (23.5)	20 (28.2)	0.489
TOAST				0.401
Larger artery disease	21 (12.4)	14 (16.1)	7 (10.1)	
Small vessel disease	49 (29.0)	23 (26.4)	26 (37.7)	
Cardioembolism	29 (17.2)	17 (19.5)	12 (17.4)	
Other determined	38 (22.5)	24 (27.6)	14 (20.3)	
Undetermined	19 (11.2)	9 (10.3)	10 (14.5)	
Initial NIHSS score	2 [0–4]	2 [0–4]	2 [1–3]	0.671
Acute thrombolysis				
t PA	7 (4.0)	4 (4.1)	3 (4.2)	0.963
IA thrombectomy	13 (7.7)	8 (8.2)	5 (7.0)	0.787
BDI	11 ± 9	10 ± 9	12 ± 11	0.274
STAI	41 ± 8	41 ± 8	43 ± 8	0.116

Results are presented as the number and percentage (% column) or mean ± SD or median [IQR]. IQR, interquartile range; SD, standard deviation; TOAST, Trial of Org 10,172 in Acute Stroke Treatment; NIHSS, National Institutes of Health Stroke Scale; ACS, anterior circulation stroke; PCS, posterior circulation stroke; BDI, Beck’s Depression Inventory; STAI, State–Trait Anxiety Inventory; t PA, Tissue plasminogen activator; IA, Intra-Arterial Thrombectomy.

Dizziness was identified in 45 patients with ACS (45.9%) and 43 patients with PCS (60.6%) (Table 2). Although dizziness tended to be more frequent in PCS, the prevalence in ACS was also substantial, affecting nearly half of the patients (*p* = 0.060). Acute reperfusion therapy was administered in a small proportion of patients (4.1% received intravenous thrombolysis and 7.7% underwent intra-arterial thrombectomy), with similar rates between ACS and PCS. Importantly, the frequency of dizziness did not differ according to thrombolytic treatment status.

**Table 2:** Comparison of patients with and without dizziness depending on the two different territories.

	ACS			PCS		
	Dizziness − (*n* = 53)	Dizziness + (*n* = 45)	*p*	Dizziness − (*n* = 28)	Dizziness + (*n* = 43)	*p*
Age (years)	63 ± 14	62 ± 13	0.724	65 ± 9	62 ± 13	0.308
Male sex	34 (64.2)	33 (73.3)	0.330	15 (53.6)	30 (69.8)	0.166
Hypertension	30 (56.6)	27 (60.0)	0.734	19 (67.9)	27 (62.8)	0.662
Diabetes mellitus	13 (24.5)	12 (26.7)	0.809	13 (46.4)	12 (27.9)	0.110
Hyperlipidemia	26 (49.1)	23 (51.1)	0.839	17 (60.7)	20 (46.5)	0.242
Atrial fibrillation	9 (17.0)	12 (26.7)	0.244	6 (21.4)	6 (14.0)	0.411
Smoking	23 (43.4)	17 (37.8)	0.573	10 (35.7)	15 (34.9)	0.943
History of previous stroke	11 (20.8)	12 (26.7)	0.491	7 (25.0)	13 (30.2)	0.632
TOAST			0.898			0.802
Larger artery disease	9 (18.8)	5 (12.8)		4 (14.8)	3 (7.1)	
Small vessel disease	12 (25.0)	11 (28.2)		9 (33.3)	17 (40.5)	
Cardioembolism	10 (20.8)	7 (17.9)		5 (18.5)	7 (16.7)	
Other determined	13 (27.1)	11 (28.2)		6 (22.2)	8 (19.0)	
Undetermined	4 (8.3)	5 (12.8)		3 (11.1)	7 (16.7)	
Initial NIHSS score	2 (0–4)	2 (0–4)	0.895	2 (0–3)	1 (1–4)	0.949
Acute thrombolysis						
t PA	2 (3.8)	2 (4.4)	0.867	0	3 (7.0)	0.153
IA thrombectomy	5 (9.4)	3 (6.7)	0.618	2 (7.1)	3 (7.0)	0.979
BDI	8 ± 8	11 ± 8	0.159	11 ± 11	12 ± 10	0.429
STAI	41 ± 9	40 ± 7	0.440	43 ± 11	42 ± 5	0.283

Results are presented as the number and percentage (% column) or mean ± SD or median [IQR]. ACS, anterior circulation stroke; PCS, posterior circulation stroke; BDI, Beck’s Depression Inventory; STAI, State–Trait Anxiety Inventory; TOAST, Trial of Org 10,172 in Acute Stroke Treatment; NIHSS, National Institutes of Health Stroke Scale.

### Clinical and imaging variables associated with dizziness in ACS and PCS

We assessed clinical factors associated with post-stroke dizziness in ACS and PCS separately (Table 3). No significant differences in age or neurological severity were observed between patients with and without dizziness. Similarly, depressive (BDI) and anxiety (STAI) symptoms did not differ by dizziness status in either group.

**Table 3:** Imaging findings of patients with and without dizziness depending on the two different territories.

	ACS			PCS		
	Dizziness − (*n* = 53)	Dizziness + (*n* = 45)	*p*	Dizziness − (*n* = 28)	Dizziness + (*n* = 43)	*p*
Acute lesion location						
Frontal cortex/subcortex	26 (49.1)	23 (51.1)	0.839	NA		
Parietal cortex/subcortex	16 (30.2)	21 (46.7)	0.094	NA		
Temporal cortex/subcortex	11 (20.8)	12 (26.7)	0.491	NA		
Deep	25 (47.2)	19 (42.2)	0.624	NA		
Insular	9 (17.0)	7 (15.6)	0.849	NA		
Temporo-occipital	NA			5 (17.9)	8 (18.6)	0.937
Thalamus	NA			4 (14.3)	11 (25.6)	0.254
Midbrain	NA			2 (7.1)	0	0.075
Pons	NA			8 (28.6)	10 (23.3)	0.615
Medullar/Cerebellum	NA			6 (21.4)	20 (46.5)	0.032
SCA	NA			4 (14.3)	7 (16.3)	
AICA	NA			0	0	
PICA	NA			6 (21.4)	17 (39.5)	
Dorsal brainstem stroke	NA			7 (25.0)	5 (11.6)	0.142
Multiple lesions	21 (39.6)	22 (48.9)	0.357	5 (17.9)	9 (20.9)	0.750
White matter hyperintensities			0.590			0.770
Fazeka 0–1	38 (71.7)	30 (66.7)		22 (78.6)	35 (81.4)	
Fazeka 2–3	15 (28.3)	15 (33.3)		6 (21.4)	8 (18.6)	
Lacune	18 (34.0)	14 (31.1)	0.764	6 (21.4)	11 (25.6)	0.689
Microbleeds	6 (11.3)	13 (28.9)	0.028	5 (17.9)	4 (9.3)	0.290
Location			0.158			0.508
Lobar	3 (5.7)	5 (11.1)		3 (10.7)	3 (7.0)	
Deep	2 (3.8)	4 (8.9)		2 (7.1)	1 (2.3)	
Both	1 (1.9)	4 (8.9)		0	0	
Number of microbleeds			0.058			0.290
1–2	6 (11.3)	11 (24.4)		5 (17.9)	4 (9.3)	
> 3	0	2 (4.4)		0	0	

Results are presented as the number and percentage (% column) NA, not available. ACS, anterior circulation stroke; PCS, posterior circulation stroke; SCA, superior cerebellar artery; AICA, anterior inferior cerebellar artery; PICA, posterior inferior cerebellar artery.

MRI findings are summarized in Table 3. In ACS, the most frequent acute lesion site was the frontal cortex and subcortex (50.0%), whereas in PCS it was the pons (25.3%). Insular involvement was observed in 16.3% of patients. The median Fazekas grade was 1 [1–2] in ACS and 1 [0–1] in PCS, with 30 (30.6%) and 14 (19.7%) showing moderate leukoaraiosis (Fazekas ≥2). CMBs were detected in 19 (19.3%) of ACS patients and 9 (12.6%) of PCS patients. Between patients with and without dizziness, those with dizziness were more likely to have microbleeds in the ACS group (28.9% vs. 11.3%; *p* = 0.028), whereas in the PCS group, dizziness was significantly associated with medullary or cerebellar involvement (46.5% vs. 21.4%; *p* = 0.032).

### Independent factors associated with dizziness

Multivariable logistic regression was performed to determine whether MRI findings significant in univariate analyses remained after adjustment for potential confounders, including age. In the ACS group, Model 1 revealed that the presence of microbleeds was independently associated with dizziness [adjusted odds ratio (aOR) = 3.19, 95% confidence interval (CI) 1.09–9.32; *p* = 0.034], and Model 2 showed that the number of microbleeds was significantly associated with dizziness (aOR = 2.38, 95% CI 1.10–5.15; *p* = 0.026) (Table 4). In the PCS group, dizziness was independently associated with medullary or cerebellar involvement (aOR = 3.13, 95% CI 1.01–9.74; *p* = 0.048) (Table 5).

**Table 4:** Multivariable analysis of factors associated with dizziness in patients with ACS.

	Model 1*		Model 2^#^	
	aOR (95% CI)	*p*	aOR (95% CI)	*p*
Age (years)	0.99 (0.96–1.02)	0.716	0.99 (0.96–1.02)	0.78
Parietal cortex/subcortex	1.38 (0.59–3.20)	0.451	1.35 (0.57–3.17)	0.48
Microbleeds	3.19 (1.09–9.32)	0.034		
Microbleeds (number)			2.38 (1.10–5.15)	0.026

Results are presented as Odds ratio and 95% confidence intervals (CIs). ACS, anterior circulation stroke. ^*^Model 1 adjusted for age, parietal cortex/subcortex, microbleeds. ^#^Model 2 adjusted for age, parietal cortex/subcortex, number of microbleeds.

**Table 5:** Multivariable analysis of factors associated with dizziness in patients with PCS.

	aOR (95% CI)	*p*
Age (years)	0.97 (0.93–1.02)	0.356
pons	1.19 (0.37–3.80)	0.765
Medullar/cerebellum	3.13 (1.01–9.74)	0.048

Results are presented as Odds ratio and 95% confidence intervals (CIs). PCS, posterior circulation stroke. Multivariable logistic regression adjusted for age, pons, medulla/cerebellum.

## Discussion

In this prospective study, we investigated the associated factors of dizziness in patients with ACS compared with PCS. We found that dizziness was common in ACS, affecting nearly half of the patients, with an incidence comparable to that of PCS. Multivariable analysis revealed that in PCS, dizziness was primarily associated with lesion topography (specifically medullary or cerebellar involvement); conversely, in ACS, dizziness was associated with the presence of microbleeds. These findings indicate that dizziness in ACS and PCS, although similarly prevalent, arises from distinct pathophysiological processes.

One notable strength of this prospective study was its focus on the characteristics and factors influencing dizziness in ACS patients. In our study cohort, over 40% of patients with ACS reported experiencing dizziness symptoms. The frequency of dizziness in ACS was higher than that described in earlier reports (4, 23–25); this discrepancy may be attributable to the early timing of questionnaire administration (median, 1 day post-event), both of which may have contributed to higher prevalence estimates (25).

The factors associated with dizziness differed significantly between anterior and posterior circulation strokes. In anterior circulation stroke, dizziness was significantly associated with the presence of CMBs. CMBs reflect chronic arteriolar injury resulting from hypertension, amyloid deposition, or ischemic gliosis, and have been associated with microstructural white matter damage. Given that intact white matter integrity is critical for efficient inter-regional brain signaling, such microvascular injury may increase vulnerability of distributed neural networks involved in balance and spatial perception (26–31).

Consequently, the presence of CMBs in patients with ACS may reflect an underlying microvascular substrate or vulnerability affecting white matter pathways, which could predispose to impaired neural network function and the clinical manifestation of dizziness (32). However, because white matter involvement was evaluated using only Fazekas grading, which did not show a significant association with dizziness, and because the number of patients with specific deep-tissue CMB involvement was relatively small, these network-level interpretations remain speculative. In the absence of tract-based or connectivity analyses, such as diffusion tensor imaging, these findings should be interpreted as a potential framework for network-level vulnerability rather than as a definitive structural mapping.

In contrast, in PCS, dizziness was primarily associated with medullary or cerebellar lesions, which are well known to directly affect neural circuits governing balance and coordination and are therefore more likely to produce overt vestibular symptoms (33). Taken together, our findings support a conceptual distinction between dizziness in anterior and posterior circulation stroke. In PCS, dizziness appears to be predominantly topographical, arising from direct injury to core vestibular structures within the brainstem and cerebellum. In contrast, dizziness in ACS may be better understood as a structural or systems-level phenomenon, reflecting vulnerability of distributed neural networks rather than focal damage to classical vestibular pathways. In this context, CMBs may serve as a surrogate marker of underlying microvascular injury or reduced white matter integrity, predisposing patients to dizziness even in the absence of overt vestibular signs.

From a clinical perspective, these observations highlight the importance of systematically assessing dizziness in patients with ACS, even when posterior circulation involvement is absent. Because dizziness in ACS may present as a subtle, non-vestibular, or poorly localizing symptom, it can be easily overlooked during routine neurological evaluation. Increased awareness and structured symptom assessment may help clinicians better recognize this frequently underappreciated manifestation, identify patients with greater cerebrovascular vulnerability, and inform more tailored post-stroke management strategies. Such an approach may ultimately contribute to improved patient comfort and long-term quality of life.

This study has several limitations. First, our study population predominantly consisted of patients with mild strokes (median NIHSS score of 2), and a substantial proportion of consecutive patients were excluded, which may introduce selection bias toward patients with less severe neurological deficits. This was unavoidable given the questionnaire-based design, which required reliable symptom reporting. Second, this was a single-center study, which may limit generalizability. Third, questionnaires were administered a median of 1 day after stroke onset, which may not fully capture the temporal evolution of symptoms. Fourth, vestibular function tests (e.g., VOG or caloric tests) were not performed. However, the primary aim of this study was not to determine the precise cause of dizziness but rather to describe the occurrence and associated clinical features after acute stroke. Fifth, the long-term prognosis of dizziness and its impact on quality of life were not assessed. Future multicenter studies with extended follow-up and objective vestibular testing are warranted. Finally, advanced imaging techniques, such as tract-based spatial statistics or functional connectivity analyses, were not performed. Accordingly, our interpretation of cerebral microbleeds as a marker of network-level impairment remains hypothesis-generating and requires validation in future studies using high-resolution connectivity imaging.

## Conclusion

Dizziness was frequent after ACS, affecting nearly half of the patients, with a prevalence comparable to PCS. In ACS, dizziness was independently associated with cerebral microbleeds, whereas in PCS it was mainly linked to medullary or cerebellar lesions. These findings indicate that post-stroke dizziness arises from distinct pathophysiological mechanisms across territories, highlighting the need for territory-specific assessment and management.

## Data Availability

The raw data supporting the conclusions of this article will be made available by the authors, without undue reservation.
