# Supplementary material for: Post-stroke dizziness in anterior vs. posterior circulation ischemic stroke
Source: Front Neurol. 2026 Feb 26;17:1742461. doi: 10.3389/fneur.2026.1742461 (PMC12979115; doi:10.3389/fneur.2026.1742461)
Supplement: Supplementary file 1 [file Data_Sheet_1.docx]

**Supplementary Table 1. Baseline Characteristics of Patients With and without dizziness survey**

|  | **Survey**  **n = 265** | **Non-survey**  **n = 711** | **p-value** |
| --- | --- | --- | --- |
| Age (years) | 64 ± 13 | 68 ± 14 | <0.001 |
| Male sex | 177 (66.8) | 447 (62.9) | 0.256 |
| Hypertension | 167 (63.0) | 471 (66.2) | 0.346 |
| Diabetes mellitus | 87 (32.8) | 251 (35.3) | 0.470 |
| Hyperlipidemia | 134 (50.6) | 340 (47.8) | 0.445 |
| Atrial fibrillation | 42 (15.8) | 140 (19.7) | 0.171 |
| Smoking | 99 (37.4) | 243 (34.2) | 0.354 |
| History of previous stroke | 71 (26.8) | 204 (28.7) | 0.557 |
| Initial NIHSS | 2 [0–4] | 3 [1–8] | <0.001 |

Results are presented as number and percent (% column) or mean ± SD or median [IQR].

IQR, interquartile range; NIHSS, National Institutes of Health Stroke Scale; SD, standard deviation

**Table S2. Clinically assessed objective neurological and vestibular signs in patients with dizziness**

|  | **ACS**  **N=45** | **PCS**  **N=43** |
| --- | --- | --- |
| **Positive neurological signs** |  |  |
| Diplopia |  |  |
| Horizontal | 0 (0.0) | 4 (11.4) |
| Vertical | 0 (0.0) | 2 (5.7) |
| Hemianopsia | 0 (0.0) | 2 (5.7) |
| Nystagmus | 0 (0.0) | 2 (5.7) |
| Ataxia (limb and/or truncal atxia) | 12 (23.1) | 11 (30.6) |

Results are presented as the number and percentage (% column) or mean ± SD or median [IQR]. ACS, anterior circulation stroke; PCS, posterior circulation stroke
